# Role of amyloid peptides in vascular dysfunction and platelet dysregulation in Alzheimer’s disease

**DOI:** 10.3389/fncel.2015.00065

**Published:** 2015-03-03

**Authors:** Ilaria Canobbio, Aisha Alsheikh Abubaker, Caterina Visconte, Mauro Torti, Giordano Pula

**Affiliations:** Department of Biology and Biotechnology, Unit of Biochemistry, University of PaviaPavia, Italy

**Keywords:** amyloid peptides, platelets, vascular cells, cerebrovascular disease, Alzheimer’s disease

## Abstract

Alzheimer’s disease (AD) is the most common neurodegenerative cause of dementia in the elderly. AD is accompanied by the accumulation of amyloid peptides in the brain parenchyma and in the cerebral vessels. The sporadic form of AD accounts for about 95% of all cases. It is characterized by a late onset, typically after the age of 65, with a complex and still poorly understood aetiology. Several observations point towards a central role of cerebrovascular dysfunction in the onset of sporadic AD (SAD). According to the “vascular hypothesis”, AD may be initiated by vascular dysfunctions that precede and promote the neurodegenerative process. In accordance to this, AD patients show increased hemorrhagic or ischemic stroke risks. It is now clear that multiple bidirectional connections exist between AD and cerebrovascular disease, and in this new scenario, the effect of amyloid peptides on vascular cells and blood platelets appear to be central to AD. In this review, we analyze the effect of amyloid peptides on vascular function and platelet activation and its contribution to the cerebrovascular pathology associated with AD and the progression of this disease.

## Alzheimer’s disease and amyloid peptides

Alzheimer’s disease (AD) is the most invalidating neurological disorder in the elderly and affects 45 million people worldwide. In parallel with population aging, AD patients are expected to rise to 115 million in 2050 worldwide (World Alzheimer Report et al., 2011).^1^ The social and economic burden of AD is high and has been calculated that in the US alone 600 billion dollars per year are spent for AD patients’ care (The Lancet Neurology et al., 2010). In the UK, dementia costs the economy more than cancer and heart disease combined (Alzheimer research UK site). Despite several promising advances in this field (Blennow et al., 2015), effective predictive markers of AD are still elusive and diagnosis relies on cognitive symptoms characteristic of the advanced stages of the disease. Brain scan and postmortem histological analysis of brain specimens remain the most reliable tools for AD diagnosis (Ikonomovic et al., 2008).

AD results in progressive loss of cognitive function and memory, which ultimately depends on extensive loss of cerebral tissue functionality linked to neuronal death (Gandy, 2005). The histological hallmarks of AD throughout the brain (mainly in the region of hippocampus and neocortex) are intracellular deposition of hyperphosphorylated form of the microtubule associated protein tau called neurofibrillary tangles (NTF) and extracellular accumulation of amyloid β peptides (Aβ peptides) in senile plaques (Bloom, 2014). Deposits of Aβ peptides are also observed in the cerebrovasculature, where they may develop cerebral amyloid angiopathy (CAA; Viswanathan and Greenberg, 2011).

**Figure 1:**
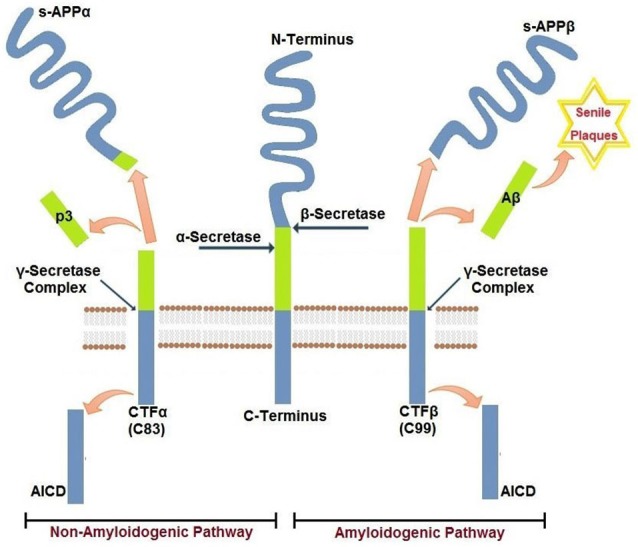
**Amyloidogenic and non-amyloidogenic pathways of APP**. Amyloid precursor protein APP is a single pass transmembrane glycoprotein. APP may be cleaved by β−γ secretases (amyloidogenic) releasing amyloid Aβ peptide(s) or by α−γ secretases (non-amyloidogenic). The two pathways are mutually exclusive and are detailed in the text.

Aβ peptides that accumulate in cerebral senile plaques and vessel walls derive from the metabolism of the larger glycoprotein called amyloid precursor protein (APP), which is a type 1 membrane glycoprotein expressed ubiquitously in the cells. APP isoform 695 is mainly expressed in neurons, whereas APP751 and APP770 that contain the Kunitz type serine protease inhibitory domain KPI are mainly expressed on peripheral cells and platelets (Van Nostrand et al., 1994). APP can be processed via two alternative pathways, amyloidogenic and non-amyloidogenic (Gandy, 2005). The amyloidogenic pathway produces Aβ peptides by the subsequent action of β- and γ-secretases. β-secretase proteolyses APP in the extracellular domain to generate soluble amyloid precursor protein β (sAPPβ) and a carboxyl terminal fragment named CTFβ (C99), which is the substrate for γ secretase to produce Aβ peptides. β secretase, or BACE, is a membrane bound aspartyl protease, for which APP is a substrate (Vassar et al., 2014). For this reason, BACE antagonists have been proposed for treatment of AD (Menting and Claassen, 2014). γ- secretase activity is contained within a molecular complex formed by the association of four essential subunits: presenilin1 or presenilin2 (either PS1 or PS2), nicastrin, presenilin-enhancer-2 (PEN-2) and anterior-pharynx-defective 1 (APH-1; Iwatsubo, 2004). The presenilins are ubiquitously expressed and represent the catalytic subunits of γ- secretase, whereas the other subunits help to stabilize the complex and to recruit the substrates to be cleaved (Smolarkiewicz et al., 2013). Alternatively, APP may be proteolysed through the non-amyloidogenic pathway, via the action of α- and γ-secretase (Buxbaum et al., 1998). α-secretase cleaves APP within the Aβ sequence, thus precluding the generation of Aβ peptides. Cleavage within Aβ sequence of APP by α-secretase generates soluble amino terminal fragments of 100–130 kDa (sAPPα) and a carboxyl terminal fragment named CTFα (C83), which is substrate for γ-secretase (Esch et al., 1990; Vingtdeux and Marambaud, 2012) to produce the non amyloidogenic peptide p3. sAPPα generated from the cleavage of APP by α-secretase shows biological functions in growth regulation and neuroprotection and, in the case of forms containing the Kunitz-type serine proteinase inhibitors KPI, in blood coagulation (Xu et al., 2007). A schematic representation of APP metabolism is presented in Figure 1.

Aβ peptides are heterogeneous short hydrophobic peptides (<5 kDa) ranging between 38 and 43 amino acids. Several different Aβ peptide species exist, but Aβ_1–40_ (Aβ_40_) is the most abundant. Aβ_40_ peptides contain 16 amino acid residues in the amino extracellular domain of APP, and 24 amino acids in the membrane-spanning domain. Aβ_42_ has two additional hydrophobic residues, Ile and Ala, and accounts for only 2–5% of Aβ peptides. Aβ_42_ peptides are more hydrophobic and fibrillogenic, have a higher aggregation potential and are the principal species deposited in the brain (Murphy and LeVine, 2010). Aβ peptides are normally generated in healthy cells (Seubert et al., 1992), but deficiencies in their clearance and/or their abnormal production and accumulation in the brain and in the cerebral blood vessels are correlated with the onset of AD. Aβ peptides in their monomeric forms are soluble, whereas their oligomerisation leads to the formation of intermolecular β-sheets and precipitation in senile plaques (Tjernberg et al., 1999). Aβ oligomers have the ability to permeabilize the plasma membrane of different cell types, which triggers a series of cellular event leading to cell dysfunction and ultimately death. This mechanism of action is common to different amyloidogenic peptides, including Aβ peptides, α-synuclein, polyglutamine and APP (Glabe, 2006).

## Vascular pathology in AD

### Amyloid and vascular hypotheses

According to the “amyloid cascade hypothesis”, (Hardy and Higgins, 1992; Hardy and Selkoe, 2002) accumulation of Aβ peptides in the brain is the central event in the pathogenesis of AD. Abnormal deposition of Aβ peptides in the brain then cause plaque and tangle formation, neuronal and vascular damage, cell loss, and finally dementia. This hypothesis is supported by the finding that the familial AD (FAD) results from mutations in genes involved in APP metabolism: *APP*, *PS1* and *PS2* (Reznik-Wolf et al., 1998). These mutations cause abnormal production of Aβ_40_ and Aβ_42_ peptides, which accumulate in the brain and in the cerebral vessel walls. FAD affects less than 5% of AD cases with autosomal dominant inheritance. Symptoms develop before the age of 65 years, and the pathology is particularly aggressive and leads to death in 5–8 years.

It is noteworthy, however, that the majority of AD patients (95%) develop the pathology after the age of 65 (Kennedy et al., 2003). This form of AD is called late onset AD (LOAD) or sporadic AD (SAD) and has a very complex etiology. The most common risk factor for LOAD/SAD is aging. A correlation has been demonstrated for the presence of the ε4 allele for the cholesterol transporter apolipoproteinE (APOE ε4) and the risk and the age of onset of AD (Michaelson, 2014). Other risk factors for LOAD/SAD are hypercholesterolemia, hypertension, Down syndrome, metabolic syndrome, diabetes, smoking and obesity (DeFronzo and Ferrannini, 1991; Gorelick, 2004; Morris et al., 2014).

The etiology of LOAD/SAD remains elusive and the role of Aβ peptides is controversial (Lee et al., 2004; Sorrentino et al., 2014). Several lines of evidence point towards a central role of early vascular dysfunction in the onset of LOAD/SAD. The “vascular hypothesis” for AD was first proposed in 1993 by De La Torre, after the observation of extensive abnormalities of cerebral capillaries in AD brain that finally results in brain hypoperfusion and reduced cerebral blood flow (de la Torre and Mussivand, 1993). In this context, it is important to note that the first case reported by Dr. Alzheimer in 1906 revealed not only the presence of senile plaques but also the presence of cerebrovascular dysfunction. In addition, Glenner and Wong firstly isolated Aβ peptides from the meningeal vessels of a LOAD/SAD patient in 1984 (Glenner and Wong, 1984). This led to the coin of the term “vascular dementia”, which indicates the loss of cognitive functions due to cerebral blood vessel alteration and poor blood supply to the brain (de la Torre, 2000; Kara et al., 2012). Vascular dementia and AD are correlated and largely overlapping phenomena (Ahtiluoto et al., 2010), and vascular dysfunctions in the brain are recognized as a contributing factor to AD (Viswanathan et al., 2009). To date, many patients with AD present vascular symptoms, including altered cerebral blood flow, damaged cerebral vasculature, and abnormal hemostasis (de la Torre, 2004a; Brundel et al., 2012). Tolppanen et al. demonstrated that AD patients, especially younger patients, have higher risk of hemorrhagic strokes (adjusted hazard ratio of 1.34; Tolppanen et al., 2013). Similarly, a recent Taiwanese population based cohort study showed that clinical diagnosis of AD is associated with considerably increased risk of stroke development (odd ratio of 1.66–1.70; Chi et al., 2013). Moreover, clinical studies indicate that asymptomatic spontaneous cerebral emboli are highly correlated with AD (Purandare and Burns, 2009) and that cerebral microinfarctions occur more likely in AD patients than in healthy controls (Brundel et al., 2012).

These observations have led to the critical question: “is AD a neurodegenerative or a vascular disorder?” (de la Torre, 2004b). Cerebral blood flow is reduced and conversely many vascular defects are present in patients with AD (Bell and Zlokovic, 2009) and vascular diseases such as atherosclerosis correlate in severity with dementia and other symptoms of LOAD/SAD (Farkas and Luiten, 2001; Roher et al., 2003; Casserly and Topol, 2004; Tibolla et al., 2010). Interestingly, vascular risk factors such as aging, hypercholesterolemia, hypertension, diabetes and obesity that predispose to atherosclerosis, stroke and cardiac disease are also associated with cerebrovascular dysfunction, which might finally results in vascular dementia and the onset of AD (Morris et al., 2014). Accumulating body of evidence gathered from human studies and animal models of AD documented that cerebrovascular dysfunction precedes the development of cognitive decline and AD pathology (de la Torre, 2010; Jellinger, 2010; Kalaria, 2010). The two-hit hypothesis has been proposed regarding AD pathogenesis, where vascular dysfunction plays a primary role in causing neurological injury. Cerebrovascular abnormalities associated to AD can result in hypoperfusion, hypoxia, focal chronic inflammation and compromised function and permeability of brain blood barrier (BBB). These injuries are exacerbated by Aβ peptides deposition, which may initiate neurodegeneration and eventually cognitive decline (Zlokovic, 2005; Grammas, 2011; Kelleher and Soiza, 2013; Li et al., 2015).

To date, it has been shown that cerebrovascular dysfunction accelerates Aβ_40–42_ production and deposition in the cerebral vasculature (Honjo et al., 2012) and facilitates the onset of CAA (Viswanathan and Greenberg, 2011). Aβ peptides are found in plasma with the levels fluctuating widely among individuals over time both in AD and in healthy control (the amount of Aβ_40_ in healthy subjects varies between 16 to 659 pg/ml, the amount of Aβ_42_ from 4 to 149 pg/ml) (Roher et al., 2009). These data are likely to be affected by the hydrophobic nature of Aβ peptides, which makes the peptides bind to a variety of plasma and membrane proteins hindering their effective measurement in biological fluids. The source of Aβ in plasma is uncertain. Some authors suggest that plasma Aβ may derive from the central nervous system through the BBB, but most authors agree and it is now well recognized that plasma Aβ derives from endothelial and circulating cells, among them platelets (Li et al., 1998; Davies et al., 2000). Circulating blood platelets are the second source of APP protein after the brain and are the major source of Aβ peptides released in plasma (Li et al., 1998). Other peripheral sources of Aβ peptides are skeletal muscle (Kuo et al., 2000; Van Nostrand and Melchor, 2001) and endothelial vascular cells (Kitazume et al., 2012). Interestingly, Aβ peptides are able to actively pass the BBB (Deane et al., 2003, 2004), and the BBB damage typical of cerebrovascular pathologies is likely to increase the exchange of Aβ peptides between cerebral and peripheral tissues, including blood. Importantly, a recent study has demonstrated that peripheral reduction of Aβ peptides is sufficient to reduce Aβ levels in the brain, which confirms the extracerebral origin of plasma Aβ peptides (Sutcliffe et al., 2011).

### AD and vascular inflammation

A number of morphological changes have been observed in the cerebromicrovascular; these include an overall decrease in intimal tight junctions (Stewart et al., 1992), basal membrane thickening (Thal et al., 2003; Kelleher and Soiza, 2013) (particularly found in areas of Aβ peptides deposition) and intimal atrophy (Farkas and Luiten, 2001; Truran et al., 2014). In AD brain, substantial morphological and functional cerebrovascular abnormalities were observed including, microvasculature irregularities and atrophy, basement membrane disruption and deposition of heparin sulfate proteoglycans, collagen IV and laminin, decreased cerebrovascular network density, endothelial cell alteration i.e., increased pinocytosis, decreased levels of mitochondria and detection of elevated endothelial cell markers VCAM-1 and E-selectin (Kalaria and Pax, 1995; Farkas and Luiten, 2001; Bailey et al., 2004; Christov et al., 2008; Zuliani et al., 2008). These vascular alterations are referred to as CAA, which associates with ischemic lesions, micro- and macro-hemorrhages, and impaired cerebral blood flow. Intimal inflammation and endothelial damage are also likely to contribute to platelet stimulation in AD patients (Borroni et al., 2002a,b). Vascular risk factors, frequently present in AD, preactivate endothelial cells and exacerbate vascular inflammation, which contributes to CAA (Zhang et al., 2013). The inflammatory proteins overexpressed in the AD cerebrovasculature are likely to have toxic effects on neurons, which could represent an important link between vascular inflammation and neuronal loss in AD (Tripathy et al., 2013). Failure in the clearance of Aβ peptides may therefore result in inflammation and neurotoxicity (Giri et al., 2000; Li et al., 2009) and CAA is likely to play a key part in brain damage and loss of cognitive skills typical of AD (Lee et al., 2014). In accordance to this hypothesis, human studies and animal models of AD document that cerebrovascular dysfunction precedes the development of cognitive decline and AD pathology (de la Torre, 2004b; Bell and Zlokovic, 2009; Xu et al., 2014).

**Figure 2:**
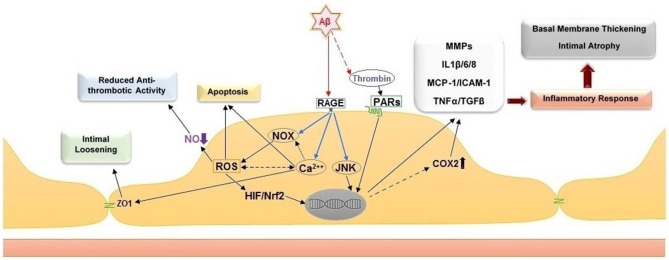
**Cellular and histological effects of amyloid peptide β on the endothelium**. Direct stimulation of the receptor for advanced glycation end products (RAGE) stimulates NADPH oxidases (NOXs), intracellular calcium increase and c-Jun N-terminal kinases (JNKs). The stimulation of NOXs and generation of reactive oxygen species (ROS) induce the activation of hypoxia-induced factor 1 (HIF) and NF-E2-related Factor 2 (Nrf2). Together with the activation of JNKs, the activity of these transcription factors leads to upregulation of pro-inflammatory genes, including cyclooxygenase 2 (COX2), metalloproteases (MMPs), interleukins 1β/6/8 (IL1β/6/8), monocyte chemoattractant protein-1 (MCP-1), intercellular adhesion molecule 1 (ICAM-1), tumor necrosis factor α (TNF α) and transforming growth factor beta (TGF-β). Additional effect of ROS increase are the reduction of nitric oxide (NO) bioavailability, which leads to pro-thrombotic endothelial cell phenotype, and apoptosis. The intracellular calcium increase leads to intimal loosening via zona occludens protein 1 (ZO-1). The accumulation of thrombin in the amyloid plaques further facilitates endothelial inflammation via stimulation of the protease-activated receptors (PARs).

Several biochemical alterations of endothelial cell physiology have been identified in response to exposure to amyloidogenic peptides (Figure 2). Amongst known inflammatory markers, cyclooxygenase 2 (COX2, a key enzyme for inflammatory reactions in platelets) expression is increased in mild cognitive impairment (MCI) and AD patients. This observation links suggestively well with several studies indicating that treatment with nonsteroidal anti-inflammatory drugs (NSAIDs) and COX2 inhibitors may reduce the risk of AD (Szekely and Zandi, 2010). Several studies indicated elevated levels of other inflammatory mediators in AD cerebral microcirculation, where endothelial cells overexpress adhesion molecules (MCP-1, ICAM-1, CAP37), and inflammatory and stress markers such as TNFα, TGF-β, interleukins (IL-1β, IL-6, IL-8) and matrix metalloproteases (MMPs; Grammas and Ovase, 2001; Grammas et al., 2002; Thirumangalakudi et al., 2006; Yin et al., 2010; Grammas, 2011). *In vitro* studies on cultured endothelial cells revealed that exposure to Aβ_40_ increased the levels of inflammatory cytokines, including IL-6, IL-1β, MCP-1, and GRO because of JNK-AP1 activation (Vukic et al., 2009). AD senile plaques also display elevated levels of thrombin (Akiyama et al., 1992). Besides its central role in hemostasis, thrombin is a factor with complex paracrine activity that mediates a wide array of cellular processes involving inflammation, neurotoxicity and angiogenesis. Thrombin release activates endothelial cells and enhances their expression of proinflammatory proteins such as ICAM-1 and MCP-1, angiopoietin-2 release, and up-regulation of αVβ3 integrin of VEGFRs expressions (Grammas and Ovase, 2001; Tsopanoglou et al., 2002). Observations obtained from *in vivo* and *in vitro* studies, showed that thrombin can cause accelerated tau protein aggregation, neuronecrosis and can incite neurotoxic effects through multiple mechanisms (Smirnova et al., 1998; Suo et al., 2003; Mhatre et al., 2004; Park and Jin, 2008; Rao et al., 2009).

### AD and redox balance in vascular cells

Another significant change in endothelial cells associated with AD is the overexpression of receptor for advanced glycation end products (RAGE). RAGE facilitates soluble Aβ peptides transport across BBB and across the cortical neurons intracellular space (Yan et al., 1996; Sasaki et al., 2001; Takuma et al., 2009). These findings suggest a role of RAGE in Aβ deposition in the cerebral intracellular space and thereby in the enhancement of AD-associated neuronal damage (Lue et al., 2001; Takuma et al., 2009). Moreover, Aβ interaction with RAGE at the BBB contributes to the up-regulation of endothelial CCR5 expression, in a dose- and time-dependent manner, via activating Egr-1 transcription factor through JNK, ERK and PI3K pathways (Li et al., 2009). The upregulation of CCR5 stimulates trans-endothelial migration of circulating T cells that express high levels of CXCR2 and MIP-1α. MIP-1α induces the opening of endothelial tight junctions (Li et al., 2009). It has been shown that increased RAGE expression facilitates the generation of reactive oxygen species (ROS) via NADPH oxidase (NOX) in cerebral endothelial cells and up regulates adhesion and pro-inflammatory molecules (Askarova et al., 2011). ROS are highly reactive molecules that promotes cell damage by direct modification of nucleic acids and proteins. One immediate consequence of increased ROS generation in cerebrovascular cells is the reduction of NO bioavailability through its transformation in the physiological oxidant peroxynitrite (NO_3_; Beckman and Koppenol, 1996). Aβ also exerts an inhibitory effect on NO signaling via binding to cell surface receptors CD36 and CD47 and inhibiting sGC/cGMP/cGK signaling pathways; thus contributing to the lack of NO signaling observed in AD (Miller et al., 2010). Because of the important antiplatelet and vasorelaxant activity of NO, its reduction in the presence of increased ROS in AD brain is likely to promote platelet activation and thromboembolic consequences. Several *in vitro* studies on Aβ peptides showed decreased NO production in endothelial cells in a dose dependent manner at Aβ concentrations of (10^−9^–10^−6^ M) and cerebrovascular endothelium desensitization to acetylcholine, which is an endothelium dependent vasodilator, at Aβ concentration of 10^−8^ M (Grammas et al., 1995; Price et al., 2001; Hayashi et al., 2009).

In addition, ROS can activate redox-sensitive transcription factors hypoxia-inducible factor 1α (HIF1 α) and nuclear factor (erythroid-derived 2)-related factor 2 (Nrf2), which modifies gene expression of endothelial cells and can ultimately lead to the up-regulation of apoptotic/inflammatory genes (Wautier et al., 2001; Fonseca et al., 2014). Accumulative body of evidence recently shows that the main culprit behind cerebrovascular dysfunction in AD is oxidative stress and ROS production mainly by NOX induced by Aβ that eventually triggers apoptosis in association with neurovascular inflammation (Cai et al., 2003; Park et al., 2005). High levels of pro-angiogenic gene expression have also been documented in AD brains using genome-wide expression profiling and includes the expression of vascular endothelial growth factor (VEGF), HIF-1α, angiopoietin-2 and thrombin (Pogue and Lukiw, 2004; Thirumangalakudi et al., 2006). This is likely to be an adaptive yet inefficient response to poor blood flow and ischemia in AD brains. In fact, AD cerebral tissue is characterized by reduced cerebromicrovascular density (Brown and Thore, 2011). Despite the increased expression of factors that promotes angiogenesis and neovascularization, several studies demonstrated an overall decrease in vasculature density in AD brains. It has been suggested that vascular-restricted mesenchyme homeobox 2 gene (MEOX2) signaling is impaired in AD patients, which interferes with the process of angiogenesis (Jellinger, 2002; Paris et al., 2004; Wu et al., 2005).

### AD and Ca^2+^ homeostasis in vascular cells

In addition to ROS, different cell populations in AD patients and animal models of the disease documented deregulation of Ca^2+^ homeostasis (Berridge, 2013; Garwood et al., 2013). Aβ has been shown to form cation permeable pores in the plasma membranes (Pollard et al., 1993) and it has been demonstrated that Aβ toxicity derives from the deregulation of Ca^2+^ homeostasis (Demuro et al., 2005). Mitochondrial Ca^2+^ overload causes mitochondrial depolarization and impairs the electron transport chain, which increases ROS generation in vascular cells (Carvalho et al., 2009). Ca^2+^ overload can directly activate NOXs (De Bock et al., 2013) and contribute to the redox stress of AD cerebral tissue. Ca^2+^ modulation seems to be critical for the onset of redox stress in endothelial cells. Aβ_40_ induces mitochondria-mediated apoptotic endothelial cell death pathway involving ER-to-mitochondria Ca^2+^ transfer, decrease of mitochondrial membrane potential, and release of pro-apoptotic factors (Fonseca et al., 2013). Recent studies showed that short-term treatment with a toxic dose of Aβ_40_ inhibits Ca^2+^ retention in the endoplasmic reticulum and enhances adenosine triphosphate (ATP)-stimulated release of intracellular Ca^2+^ (Fonseca et al., 2014). Importantly, Aβ-RAGE interactions in cultured endothelial cells increase intracellular Ca^2+^ and alter tight junction proteins through the Ca^2+^-calcineurin pathway (Kook et al., 2012). This phenomenon is responsible for the reduction of zonula occludin-1 (ZO-1) expression and increased MMP secretion observed in cultured endothelial cells and can ultimately be the cause of enhanced MMP secretion and loss of vascular integrity in the vicinity of Aβ plaques in animal models of AD (Kook et al., 2012). The resulting Aβ-dependent alteration in the integrity of the BBB may play a significant role in the transport and accumulation of Aβ peptides from the circulation to the brain parenchyma.

## Platelet pathology in AD

### APP and Aβ peptides in platelets

The bidirectional correlations between AD and vascular diseases points to an important role for non-neuronal cells and extraneuronal generation of Aβ peptides in the development of AD. Circulating platelets, which are anucleate cells responsible for hemostasis and thrombosis, are good candidates for the link between vascular diseases and AD. Platelets express mainly the APP isoforms 751 and 770. These isoforms contain the 56 amino acids long Kunitz type serine protease inhibitors domain KPI, which is known to inhibit serine proteases including those of the coagulation cascade (Van Nostrand et al., 1989; Xu et al., 2005, 2007). In addition, platelets express all the enzymatic machinery responsible for APP metabolism and for the generation of Aβ peptides (Evin et al., 2003; Catricalà et al., 2012). In physiological conditions, platelets metabolize APP through the non-amyloidogenic pathway: α-secretase is activated by Ca^2+^ and calmodulin (Canobbio et al., 2011) and releases soluble APPα. To a lesser extend platelets also release Aβ peptides. The main species of Aβ released from activated human platelets is Aβ_40_, while the predominant form in neuronal plaques is Aβ_42_. Immunoassay analysis has revealed that the inactivated platelets had an average of 84 ng/g tissue of Aβ_40_ and activated platelets contained an average of 57 ng/g tissue, while in both cases there were very low levels of Aβ_42_: 1.6 ng/g tissue and 1.7 ng/g tissue, respectively (Kokjohn et al., 2011). APP fragments and Aβ peptides are stored in platelet α-granules as revealed by immune electron microscopy (Li et al., 1994) and sucrose density gradient (Van Nostrand et al., 1990), and are released upon stimulation with physiological agonists (Li et al., 1994). Increasing evidence suggests that released Aβ peptides are able to activate platelets and promote platelet adhesion and aggregation (Shen et al., 2008a,b; Canobbio et al., 2013, 2014). This eventually results in enhanced thrombus formation *in vitro* and *in vivo* (Gowert et al., 2014; Sonkar et al., 2014). On the contrary, soluble APP fragments have been shown to regulate thrombosis and hemostasis *in vivo* (Xu et al., 2005, 2007).

The relatively abundant concentration of APP in platelets (9300 copies/platelet) (Rowley et al., 2011) and the predominant presence in platelets of the KPI containing isoforms suggest that APP may have a physiological role, mainly in events associated with coagulation (Van Nostrand et al., 1992; Xu et al., 2005). A minority (10%) of APP is expressed as an intact glycoprotein on the platelet surface where it may act as a receptor on the platelet surface (Kang et al., 1987; Li et al., 1994). Fibrillar Aβ but not unassembled Aβ, specifically binds to APP (Lorenzo et al., 2000; Van Nostrand et al., 2002; Sola Vigo et al., 2009). APP also can bind to sulfated proteoglycans, laminin, collagen and integrin like receptor, suggesting a role in cell-cell and cell- matrix interactions (Breen et al., 1991; Ghiso et al., 1992; Milward et al., 1992; Beher et al., 1996; Williamson et al., 1996; Verdier and Penke, 2004). Moreover several studies have reported that treatment of neuronal cell with monoclonal antibody 22C11 directed toward the N terminal domain of APP can stimulate G protein activity (Okamoto et al., 1995) and/or cause the dimerization of APP (Scheuermann et al., 2001). Notably, APP metabolism resembles that of Notch (Nakayama et al., 2011): in the canonical Notch signaling pathway, γ-secretase cleaves Notch in the plasma membrane and release an intracellular domain that shows activity in the nucleus through binding to transcription factors. Since platelets are anucleate cellular fragments this consideration deserve further investigation.

APP is proteolytically cleaved during platelet activation (Li et al., 1998; Evin et al., 2003). The released soluble APP fragment that contain KPI domain was firstly identify as Protease Nexin 2 PN-2, a chymotrypsin inhibitor and regulator of blood coagulation (Van Nostrand et al., 1989). More recently it has been shown by the same authors that PN-2 coincide with soluble APP fragment containing KPI (Van Nostrand et al., 1992). Soluble APP inhibits the activity of the blood coagulation factors IXa, XIa, and Xa, and, to a lesser extent, of factor VIIa-tissues factor complex (Smith et al., 1990; Van Nostrand et al., 1992; Schmaier et al., 1995; Scandura et al., 1997) and may therefore play a role in the coagulation cascade, thus modulating hemostasis following vascular injury by limiting thrombosis. Xu et al. generated specific transgenic mice which expressed human APP770 in platelets, under the control of rPF4 promoter. Transgenic mice express 2 fold higher APP770 amount in platelets, but not in other analyzed tissues. They investigated the APP function comparing Tg-rPF4/APP mice with wild type mice and with mice which do not express APP at all (APP KO) and demonstrated that KPI-containing forms of APP regulates cerebral thrombosis *in vivo* in a carotid artery thrombosis and in experimental intracerebral hemorrhage (Xu et al., 2005, 2007). In accordance to this, recombinant soluble APP has been shown to inhibit platelet aggregation and secretion induced by ADP and adrenaline *in vitro* (Henry et al., 1998).

### Aβ peptide-dependent activation of platelets

**Figure 3:**
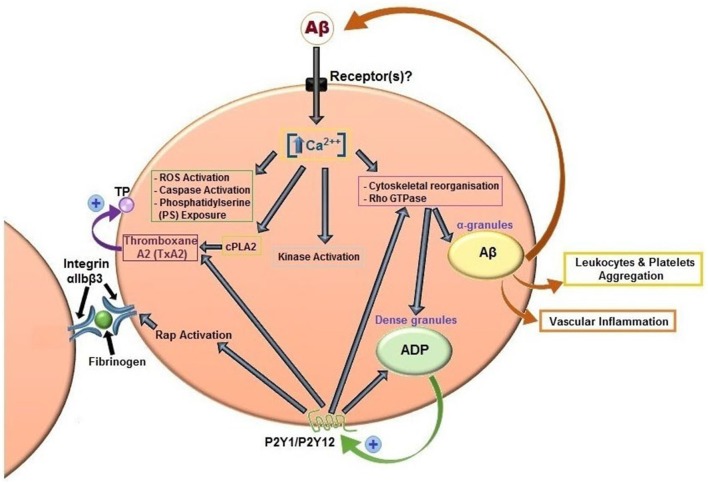
**Aβ peptides-induced platelet activation**. Aβ peptides present in plasma activate platelets inducing activation of PLC/PKC and intracellular Ca^2+^ movement, granule secretion, kinase activation, rap1b mediated-integrin activation and aggregation. Aβ peptides released from α-granules, ADP released by dense granules, and formation of TxA_2_ reinforce platelet activation. Aβ peptides also promotes ROS formation, caspase activation and membrane scrambling. Activated platelets recruit leukocytes and promotes vascular inflammation.

It has been widely demonstrated by several authors that Aβ peptides are able to activate platelets (Figure 3). The first study date back to 2007 and analyzed the effects of misfolded proteins on platelet activation. Herzenik et al. showed that proteins with amyloid properties, including Aβ peptides, activate platelets. Fibrillar Aβ peptide induces platelet aggregation through the scavenger receptor CD36 and GPIbα, and activation of intracellular signaling pathways involving p38MAPK, cyclooxygenase1 (COX1) and thromboxane A2 production (Herczenik et al., 2007). More recently, the ability of amyloid peptides to activate platelets has been investigated using the synthetic peptide Aβ_25–35_. This undecapeptide is located in the intermembrane domain of APP and retains the toxic properties of the entire peptide representing the biologically active region of Aβ (Kang et al., 1987). Aβ_25–35_ toxicity resides in the ability to form oxygen radicals, nitric oxide and to disrupt calcium homeostasis (Kaminsky et al., 2010). In preliminary studies, Aβ_40_ and Aβ_25–35_ showed equal potencies in inducing platelet activation (Shen et al., 2008a). Exogenous Aβ_25–35_ (1–2 μM) potentiated agonist-induced platelet aggregation induced by collagen and ADP, and at higher concentrations (2–10 µM) Aβ_25–35_ itself directly promotes aggregation, and activation of intracellular signaling pathways through thrombin receptor PAR1 activation of p38 MAPK and cytosolic PLA_2_, and TxA2 formation (Shen et al., 2008a). In addition, the same authors demonstrated that Aβ_25–35_ stimulates PLC/PKC activation and Ca^2+^ mobilization (Shen et al., 2008b). Aβ_25–35_ also induced the activation of small GTPase RhoA and phosphorylation of myosin light chain kinase, which leads to cytoskeletal reorganization and results in shape change, granule release, integrin activation and clot retraction. An essential role for Ca^2+^ and ADP in Aβ -induced platelet activation and thrombus formation has been recently demonstrated by our group (Canobbio et al., 2014). We have demonstrated that the first event in Aβ-induced platelet activation is the increase of intracellular Ca^2+^ concentration. Our results confirmed and extended previous observations by Galeazzi et al., on Aβ_25–35_-induced intraplatelet Ca^2+^ mobilization (Galeazzi et al., 2000). Aβ-induced intracellular Ca^2+^ movements rely on the presence of extracellular Ca^2+^, this result is in line with the evidence that Aβ is able to form cation permeable pores in the plasma membranes (Pollard et al., 1993) and that Aβ toxicity derives from the deregulation of Ca^2+^ homeostasis (Demuro et al., 2005). Intracellular Ca^2+^ increase in platelets promotes acto-myosin cytoskeletal reorganization, granule secretion and ADP release. Once released ADP is able to reinforce and promote platelet activation in term of phosphorylation of selected signaling proteins, mainly the non receptor tyrosine kinase Syk, PI3K, PKC and MLC, activation of the small GTPase Rap1b, inside-out activation of integrin α*_IIb_*β_3_, and finally aggregation (Canobbio et al., 2014).

We have demonstrated that platelets are also able to adhere and spread over immobilized Aβ_25–35_ as well as Aβ_40_ and Aβ_42_, to accelerate platelet adhesion over collagen (Canobbio et al., 2013; Gowert et al., 2014), and to increase platelet spreading over fibrinogen (Sonkar et al., 2014). Interestingly, platelets adhere to Aβ peptides independently of ADP but are able to activate several intracellular signaling pathways associated with platelet stimulation dependently on the release of ADP. Aβ peptides also enhance adhesion to collagen under shear (Canobbio et al., 2014) and potentiates thrombus formation *in vivo*, as revealed in a mouse model of pulmonary thromboembolism (Sonkar et al., 2014). The effect of Aβ in promoting platelet activation *in vivo* was confirmed in injured carotid artery model (Gowert et al., 2014). More interestingly, the effect of amyloid peptides was analyzed in APP transgenic mice. APP23 and APP Dutch mice are known to develop CAA upon aging and to deposit amyloid peptides in the cerebral vessel walls. Gowert et al. analyzed brain of these transgenic mice for Aβ deposition and platelet recruitment and demonstrated that platelets adhere to vascular amyloid plaques with time and that sustained platelet recruitment may lead to full occlusion of the vessel (Gowert et al., 2014).

Aβ peptides regulate phosphatidylserine exposure, micro-particles production and caspase activation, suggesting a transition of platelets from activation to apoptosis (Gowert et al., 2014). Recently, it has been demonstrated that mitochondrial respiration is increased in Aβ-stimulated platelets (Sonkar et al., 2014). In addition, ROS generation in platelets is significantly increased by exposure to Aβ peptides (Gowert et al., 2014). Moreover, cytochrome c oxidase, an enzyme that belongs to the complex IV of the respiratory chain, has been observed to be diminished in platelets and hippocampal mitochondria of AD patients (Bosetti et al., 2002). This result was confirmed by other studies, and is correlated with increased ROS formation in AD platelets (Cardoso et al., 2004). Taken together, these studies point toward an increased oxidative stress in AD patients. There is therefore the possibility that Aβ peptides stimulate/increase platelet activation by inducing a state of redox stress, which has been shown to increase platelet responsiveness. For example, O2—has been suggested to stimulate platelet hyperactivity in anoxia/reoxygenation conditions (Leo et al., 1997) and hypercholesterolemia (Stokes et al., 2007). The dependence of platelet activation on ROS generation explains the inhibition of platelets and the anti-thrombotic effect of antioxidants (Freedman, 2008) or ROS-generating enzyme inhibitors (Vara et al., 2013).

Table 1 summarize the effects of Aβ peptides on platelet activation.

**Table 1:** **Principal molecular effects of amyloid peptides on platelets**.

Amyloid peptide	Molecular effects on platelets	References
**Aβ_1–40_**	promotes platelet aggregation	Herczenik et al. (2007)
	promotes ROS formation, caspase activation, annexin V exposition and membrane scrambling	Gowert et al. (2014)
	promotes platelet adhesion under static and dynamic flow conditions	Canobbio et al. (2013), Gowert et al. (2014)
	increases platelet adhesion in an injured carotid artery model	Gowert et al. (2014)
	modulates soluble A**β** into fibrillar A**β**	Gowert et al. (2014)
	recruits platelets to vascular amyloid plaques	Gowert et al. (2014)
**Aβ_25–35_**	induces platelet adhesion in static conditions	Canobbio et al. (2013)
	fastens platelet spreading over collagen	Canobbio et al. (2013)
	increases platelet spreading over fibrinogen	Sonkar et al. (2014)
	increases adhesion to collagen under shear	Canobbio et al. (2014), Sonkar et al. (2014)
	promotes platelet aggregation	Shen et al. (2008b), Canobbio et al. (2014), Sonkar et al. (2014)
	potentiates platelet aggregation induced by collagen and ADP	Shen et al. (2008a)
	promotes Ca^2+^ mobilization and granule secretion	Galeazzi et al. (2000), Canobbio et al. (2014), Sonkar et al. (2014),
	activates PLC and PKC	Shen et al. (2008b)
	activates Syk, PI3K/Akt, MAP kinases	Canobbio et al. (2013, 2014)
	induces RhoA and Rap1b activation	Canobbio et al. (2014), Sonkar et al. (2014)
	induces Rap1b and integrin activation	Canobbio et al. (2014), Sonkar et al. (2014)
	triggers hydroxyl radical formation	Shen et al. (2008b)
	induces clot retraction	Sonkar et al. (2014)
	shortens platelet plug formation in mesenteric venules in mice	Shen et al. (2008b)
	induces thrombus formation in a model of pulmonary thromboembolism	Sonkar et al. (2014)

### Animal models of AD and platelet and vascular (dys)functions

Since the formulation of the “amyloid cascade hypothesis” and the discovery of mutations correlated with FAD, several AD transgenic mice have been developed to study the pathophysiological role of APP and Aβ peptides. In most cases, AD transgenic mice express mutant human *APP* or *PS1/PS2* genes (one single or more mutations correlated with AD onset in FAD). In addition, mice that do not express APP have been generated to investigate the physiological role of APP in cellular signaling. The number of murine model for AD is exponentially growing and for an extensive list of AD model mice we suggest to refer to http://www.alzforum.org/research-models. These mice have been extensively studied as a model for the onset of AD, and to investigate the mechanism underlying amyloid deposition in brain parenchyma and cerebral vessels, their correlation with cognitive impairment, and to develop new therapeutic strategy. However, only few of these murine models have been utilized for analysis of possible peripheral platelet and vascular dysfunctions. The prothrombotic phenotype observed in AD patients and related to pre-activated platelets has been demonstrated in AD transgenic mouse models. Recently, Jarre et al. analyze platelet activation in APP23 mice which carry human APP751 containing the Swedish (KM670/671NL) under the neuronal Thy promoter. These mice deposit Aβ peptides in the brain parenchyma and in the cerebral vessel walls developing CAA upon aging. In this study the authors have demonstrated that platelets of aged Alzheimer transgenic mice APP23 are in a pre-activated state and respond with enhance platelet activation upon stimulation. This finally results in a pro-thrombotic phenotype with altered hemostasis and increased thrombus formation *in vivo* (Jarre et al., 2014).

Not only platelets, but also cerebrovasculature is activated in AD transgenic mice. Grammas et al. analyzed two transgenic AD animal models: AD2576APPSwe and La Ferla3xTG which overexpressed Aβ in the brain and demonstrated that brain endothelial cells overexpressed Aβ, thrombin, tumor necrosis factor α, interleukin-1β and interleukin 6 and MMP9 (Grammas et al., 2014). Inflamed endothelial cells also release APP770 (Kitazume et al., 2012). Aβ and thrombin expressed from endothelial cells in turn strengthen platelet activation thus reinforcing chronic inflammation and thrombus formation.

### Anti platelet therapies and AD

Aβ peptides released by platelets and endothelial cells result in platelet activation and vascular inflammation with dangerous consequences for the progression of AD. With this background, it seems reasonable that the use of antiplatelet agents and/or NSAIDs may slow down AD. We have previously reported in this review that treatment with COX2 inhibitors may reduce the risk of AD (Szekely and Zandi, 2010). Epidemiological studies in the past have suggested that patients taking NSAIDs or aspirin display some level of protection from AD (Henderson et al., 1997; Aisen et al., 2000; Broe et al., 2000; in ’t Veld et al., 2002; Thal et al., 2005; ADAPT Research Group et al., 2007). Aspirin is the most common anticoagulant used in prevention of stroke and myocardial infarction (Yeung and Holinstat, 2012; Zhang et al., 2013). Aspirin is an irreversible inhibitor of cyclooxygenase 1 that catalyzes the synthesis of an important platelet agonist thromboxane A2 (Loll et al., 1995). At high doses (5 g per day) aspirin inhibits prostaglandin production by white blood cells and has therefore anti-inflammatory action, whereas at low doses (75 mg) per day has mainly anti platelet activity. Aspirin is commonly used in the treatment of vascular dementia (Molnar et al., 1998). The AD 2000 collaborative group analyzed for 3 years the effect of low dose aspirin daily administration on 156 AD patients compared to 154 AD patients who did not take aspirin. The outcomes evaluated were cognition and functional ability. The results showed no benefits of aspirin on the progression of AD in the analyzed subjects. On the contrary, 8% of patients taking aspirin had serious bleed and 2% of patients in the aspirin group had fatal cerebral bleed (AD2000 Collaborative Group et al., 2008). These results advice against the use of aspirin in AD, since the risks overweigh any benefits. However, more studies on aspirin or other antithrombotic drugs are necessary to understand the potential of these drugs in the treatment of AD. It is conceivable that the administration of antithrombotic or anti-inflammatory drugs is not effective on AD when the pathology is overt, but may be important in prevention of the disease in individuals at risk.

### Platelet abnormalities in AD

Many alterations have been observed in platelets from AD patients compared to age matched control subjects, but attention must be taken when considering the data found in the literature. The most important and reliable changes observed in platelets from AD patients are reduced APP ratio, alteration of α- and β-secretase expression or activity that result in abnormal APP metabolism through the amyloidogenic pathway, and enhanced platelet activation. In addition, changes in expression and/or activity of Monoamine oxidase B (MAO-B), cytochrome c oxidase, and cicloxygenase2 have been reported in platelets isolated from AD patients. For a comprehensive and exhaustive analysis of alterations in platelets from AD patients refer to the recent paper by Veitinger et al. (2014).

#### Altered APP ratio

APP is present on plasma membrane as an intact glycoprotein of about 110/130 kDa and as soluble fragments of different length in platelet α-granules. Based on the electrophoretic mobility of anti-APP antibody 22C11-immunoreactive bands it is possible to reveal at least two isoforms of APP in platelets: a higher band at 130 kDa and a lower band at about 106/110 kDa. The difference in electrophoretic mobility between the 130 kDa and the 106–110 kDa bands has been attributed to the presence or absence of KPI, respectively. The APP upper to lower band ratio is lower in platelets from patients affected by MCI and SAD compared to platelet prepared from control subjects and patients affected by other kind of dementia (Di Luca et al., 2000; Borroni et al., 2005, 2010). The altered APP ratio in platelets shows a positive and specific correlation to the progression of the disease. This correlation is also present in preclinical stages of AD, suggesting that this can be as a useful biomarker for AD.

#### Altered secretase activation and APP metabolism

Platelets metabolize APP preferentially through non-amyloidogenic α-secretase pathway. In platelets from AD patients, the expression of α-secretase candidate ADAM10 is decreased compared to healthy subjects (Colciaghi et al., 2002; Tang et al., 2006). On the other hand, an increased β-secretase activity has been indirectly shown by a decreased ratio of its 37/56 kDa fragments (Colciaghi et al., 2004; Tang et al., 2006). The increased β-secretase activity was also demonstrated directly by enzymatic assay in the early stage of AD and MCI patients compared to control (Liu et al., 2007; Johnston et al., 2008), and by increased level of β-secretase product sAPPβ (Colciaghi et al., 2004). Taken together, this switch from non-amyloidogenic to amyloidogenic in the APP pathway is likely to be responsible for the increase in platelet-derived Aβ peptides generation.

#### Monoamine oxidase-B activity

MAO-B is responsible for the degradation of neurotransmitters in the nervous system (e.g., dopamine) and expressed in platelets (Paasonen et al., 1964). Several studies independently described increase in MAO-B expression and activity in brain and platelets of AD patients (Adolfsson et al., 1980; Bongioanni et al., 1997; Mészáros et al., 1998). The molecular causes of this change are currently unknown.

#### Platelets are preactivated in AD patients

More than ten years ago, Sevush et al analyzed platelet activation in 91 patients with probable AD and 40 age-matched control subjects. Groups were compared for percentage of circulating platelet aggregates, surface expression of P-selectin (α granule marker), formation of leukocyte-platelet complexes, and presence of circulating platelet microparticles. The results showed a significant increase in platelet aggregates, leukocyte-platelet complexes and P-selectin expression in resting platelets of AD patients demonstrating that platelets of patients with AD exhibit greater basal activation than those of controls (Sevush et al., 1998). More recently, Stellos et al. demonstrated that integrin α*_IIb_*β_3_ is activated and P-selectin is expressed on plasma membrane in unstimulated platelets from AD subjects compared to control (Stellos et al., 2010; Laske, 2012). More interestingly, in one-year follow up study platelet activation parameters correlate with the rate of cognitive decline and anti-platelet therapy reduced cognitive decline in AD patients (Laske et al., 2010; Sakurai et al., 2013). As we previously reported, activated platelets have been shown to adhere to cerebral vascular deposits of Aβ in AD transgenic mice models, which are known to develop CAA upon aging. Sustain platelet recruitment to vascular amyloid plaques results in occlusion of the vessels (Gowert et al., 2014). The same authors also demonstrated that platelets are able to process soluble synthetic Aβ into fibrillar Aβ increasing its neurotoxicity in culture, which suggests an active role of platelets in promoting neuronal death in AD. Platelet localization at the site of cerebrovascular injury and in CAA lesions has been observed in several other studies, which demonstrated localization of activated platelets with deposit of Aβ peptides (Roher et al., 2009). This is likely to play an important role in the establishment of a vicious circle of platelet activation, Aβ release and neuronal cell death. Once activated, platelets can release Aβ_40_ in the circulation, increasing its local concentration and contributing to accumulation of amyloid peptides in the vessel of the cerebrovasculature. Amyloid accumulation also induces microvascular inflammation (Grammas and Ovase, 2001). Platelet deposition in the cerebral microvasculature may account for the hemostatic abnormalities observed in AD (Wilkerson and Sane, 2002).

#### Higher levels of “coated” platelets

Another important parameter of platelet activation is the presence of increased levels of coated platelets in AD patients. Coated platelets are a subset of activated platelets observed upon dual-agonist stimulation with collagen and thrombin characterized by high pro-coagulant activity (Dale, 2005). Prodan et al. demonstrated that coated platelets expressed high levels of full-length APP on their surface compared to single agonist-stimulated platelets (Prodan et al., 2006). Coated platelets are increased in early stages of AD, are elevated in patients with amnestic as compared to nonamnestic MCI, and their numbers correlate with disease progression in AD (Prodan et al., 2007, 2008). Coated platelets are also altered in patients with cerebrovascular disease and have been show to potentiate inflammation. These data suggest a role of coated platelets as a sensitive biomarker of AD (Prodan et al., 2009).

#### Coagulation abnormalities in AD

Besides platelet hyper activation, coagulation is also impaired in AD patients. In particular it has been observed that Aβ is able to bind fibrinogen and that fibrin clot formed in the presence of Aβ are more stable and more resistant to degradation during fibrinolysis (Ahn et al., 2010; Cortes-Canteli et al., 2010). After BBB alterations fibrinogen may deposit to brain blood vessel and accumulate in CAA and parenchyma in AD patients, and in AD mouse models (Paul et al., 2007). This could finally results in altered cerebral blood flow and worsening of the pro-thrombotic phenotype in cerebral microvasculature of AD patients.

## Conclusions and future directions

AD is associated with platelet and vascular abnormalities. Our understanding of the mechanisms underlying thrombosis and angiopathy associated with AD is growing exponentially. Several molecular mechanisms that explain microvascular alterations associated with AD have been undercovered. Although the role of tau protein in neuronal death and cognitive ability loss is also central in AD development, the accumulation of amyloid peptides is closely related to the vascular abnormalities associated with this disease. Therefore, this was the subject of this literature review.

Future challenges in this field will be to understand fully the relevance of the microvascular symptoms of AD in dementia development and to target them in order to improve the management of this debilitating disease. This will require a truly multidisciplinary effort with basic scientists and clinicians from different areas of research (i.e., neuroscience, cardiovascular sciences and cell biology) collaborating towards the clarification of the complex etiology of AD. Biomedical research into the vascular aspects of AD provides new hopes for early disease diagnosis and the development of novel clinical strategies to reduce the rate of cognitive function loss following diagnosis.

## Conflict of interest statement

The Guest Associate Editor Francesco Moccia declares that, despite being affiliated to the same institution as authors Ilaria Canobbio, Aisha Alsheikh Abubaker, Caterina Visconte, Mauro Torti and Giordano Pula, the review process was handled objectively and no conflict of interest exists. The authors declare that the research was conducted in the absence of any commercial or financial relationships that could be construed as a potential conflict of interest.

## Footnotes

^1^http://www.alz.co.uk/
